# EASIX for Prediction of Outcome in Hospitalized SARS-CoV-2 Infected Patients

**DOI:** 10.3389/fimmu.2021.634416

**Published:** 2021-06-23

**Authors:** Thomas Luft, Clemens-Martin Wendtner, Florentina Kosely, Aleksandar Radujkovic, Axel Benner, Felix Korell, Lars Kihm, Matthias F. Bauer, Peter Dreger, Uta Merle

**Affiliations:** ^1^ Department of Internal Medicine V, University of Heidelberg, Heidelberg, Germany; ^2^ Munich Clinic Schwabing, Academic Teaching Hospital, Ludwig-Maximilians University (LMU), Munich, Germany; ^3^ Medical Department B, Hospital Ludwigshafen, Ludwigshafen, Germany; ^4^ Division of Biostatistics, German Cancer Research Center, Heidelberg, Germany; ^5^ Department of Internal Medicine I, University of Heidelberg, Heidelberg, Germany; ^6^ Institute of Laboratory Diagnostics, Hygiene and Transfusion Medicine, Hospital Ludwigshafen, Ludwigshafen, Germany; ^7^ Department of Internal Medicine IV, University of Heidelberg, Heidelberg, Germany

**Keywords:** endothelial activation and stress index, EASIX, SARS-CoV2 (COVID- 19), suppressor of tumorigenicity 2 (ST2), soluble thrombomodulin, angiopoietin-2 (Ang-2), prediction of outcome

## Abstract

**Background:**

The coronavirus disease 2019 (COVID-19) is caused by severe acute respiratory syndrome coronavirus-2 (SARS-CoV-2) and has evoked a pandemic that challenges public health-care systems worldwide. Endothelial cell dysfunction plays a key role in pathophysiology, and simple prognosticators may help to optimize allocation of limited resources. Endothelial activation and stress index (EASIX) is a validated predictor of endothelial complications and outcome after allogeneic stem cell transplantation. Aim of this study was to test if EASIX could predict life-threatening complications in patients with COVID-19.

**Methods:**

SARS-CoV-2-positive, hospitalized patients were enrolled onto a prospective non-interventional register study (n=100). Biomarkers were assessed at hospital admission. Primary endpoint was severe course of disease (mechanical ventilation and/or death, V/D). Results were validated in 126 patients treated in two independent institutions.

**Results:**

EASIX at admission was a strong predictor of severe course of the disease (odds ratio for a two-fold change 3.4, 95%CI 1.8-6.3, p<0.001), time to V/D (hazard ratio (HR) for a two-fold change 2.0, 95%CI 1.5-2.6, p<0.001) as well as survival (HR for a two-fold change 1.7, 95%CI 1.2-2.5, p=0.006). The effect was retained in multivariable analysis adjusting for age, gender, and comorbidities and could be validated in the independent cohort. At hospital admission EASIX correlated with increased suppressor of tumorigenicity-2, soluble thrombomodulin, angiopoietin-2, CXCL8, CXCL9 and interleukin-18, but not interferon-alpha.

**Conclusion:**

EASIX is a validated predictor of COVID19 outcome and an easy-to-access tool to segregate patients in need for intensive surveillance.

## Introduction

Since the first cases of SARS-CoV-2 infection reported in Hubei, China, in December 2019, the virus has spread worldwide causing a still unrestrained pandemic with millions of infections and hundreds of thousands of virus-associated deaths (https://coronavirus.jhu.edu/data/new-cases). In most cases, the disease caused by SARS-CoV-2 (COVID-19) follows a mild or moderate cause with symptoms of upper airway infections, fever, fatigue, anosmia, hypogeusia, and diarrhea (1, 2). Yet, severe courses resulting in acute respiratory distress syndrome (ARDS), sepsis, hypercoagulation, myocardial injury and multi-organ failure are not uncommon and frequently require aggressive management on an intensive care unit (3). Elderly male patients with pre-existing cardio-vascular conditions have highest risk of severe morbidity and fatal outcome (4–6), however, there is an eminent heterogeneity of clinical courses, and even children may suffer from severe complications (7, 8). Given the considerable variability of clinical courses and the huge challenge of the COVID-19 pandemic to clinical resources, a reliable and readily available biomarker for early prediction of severity of COVID-19 is urgently needed in order to assign hospital resources most efficiently.

A key role for endothelial cells in the pathophysiology of ARDS, multi-organ failure and mortality associated with COVID-19 has been postulated (9–12). There is good morphological evidence for endothelial cell infection and endotheliitis in COVID-19 disease (11, 13), mediated by viral binding to the receptor for angiotensin converting enzyme 2 (ACE2) (14). These observations are in line with clinical and serological findings suggesting that endothelial activation and damage may play a central role in the pathogenesis of COVID-19-associated complications (6, 15). Specifically, there is evidence that in COVID 19, endothelial inflammatory cytokines including Angiopoietin-2 and CXCL8 enhance vascular leakage and recruit activated neutrophils, respectively (16), and that dysfunctional interaction with platelets activates coagulation and complement pathways (12). In fact, the clinical presentation of severe COVID-19 is generally consistent with the presence of microangiopathy (elevated LDH and d-dimers, complement activation, decreased platelets and renal impairment) which may predispose patients to thrombotic disease and micro-infarcts promoting multi-organ failure (9, 17, 18).

Beyond this background, we have tested if the EASIX (Endothelial Activation and Stress Index) might help to predict the clinical course of COVID-19. We developed EASIX as a simple score based on readily available routine parameters (LDH, creatinine, platelet count) in order to predict endothelial complications after allogeneic stem cell transplantation. We initially wanted to understand why patients died from immune mediated complications, such as graft-versus-host disease (GVHD), despite a large variety of readily available immunosuppressant drugs. We found that a progressive endothelial damage, i.e. transplant-associated microangiopathy (TAM), was present in most lethal courses of acute GVHD (19, 20). TAM is characterized by high LDH, high creatinine and low platelet counts, amongst others (21). We wondered if high LDH and creatinine together with low platelets (that is high EASIX) could predict these endothelial complications earlier than the accepted diagnostic criteria. Indeed, EASIX measured at onset of acute GVHD, on the day of transplantation, and even before starting the conditioning therapy for allogeneic stem cell transplantation predicted risk of mortality, as well as endothelial complications such as sinusoidal obstruction syndrome/veno-occlusive disease (SOS/VOD) and early fluid overload (22–26). EASIX also associated with mortality of lower and intermediate risk patients with myelodysplastic syndromes (27), and with mortality of multiple myeloma patients (28). EASIX is therefore a validated marker of endothelial risk both in immune mediated and malignant diseases.

Cytokines associating with EASIX and outcome of post-transplant complications include ANG2, sCD141, ST2 (19, 20, 29), CXCL9 (30) and IL18 (31, 32). Interferon-alpha represents an early but transient immune response to viral infections that appeared deficient in COVID-19 patients (33). ANG2 and other endothelial serum markers were already shown to predict severe clinical courses of COVID-19 (16, 34).

The endothelial association of COVID-19 associated complications led us to investigate EASIX together with endothelial and immune markers in COVID-19 patients admitted to the hospital. For this purpose, we performed a prospective non-interventional study and validated it retrospectively on independent datasets. The results suggest that EASIX appears to be valuable for segregating patients in need for intensive surveillance from those with an uneventful clinical course. In addition, we provide further evidence for endothelial involvement in COVID-19 pathogenesis delineating cytokine profiles associated with courses of different severity.

## Patients and Methods

### Study Design and Data Collection

Eligible for the prospective non-interventional study conducted at the University of Heidelberg were all patients who were admitted for symptomatic SARS-CoV-2 infection between February 28^th^ and May 2^nd^, 2020, and had consented to study participation. Primary endpoint was severe course of the disease defined as mechanical ventilation and/or death of any cause (V/D). Symptomatic SARS-CoV-2-positive patients from the Munich Clinic Schwabing (n=88) and Ludwigshafen Hospital (n=38) admitted during the same time period constituted the validation cohort (n=126). Written informed consent according to the Declaration of Helsinki was obtained for all patients and the local Ethics committees had approved data collection and analysis (reference numbers: S-771/2020, S-148/2020, 20-265, and 202-14949, respectively). In all centers, patients were tested for SARS-CoV-2 infection following local guidelines (https://www.muenchen-klinik.de/covid-19-share/#c57673) and in accordance with the latest recommendations of the Robert Koch Institute:

(https://www.rki.de/DE/Content/Kommissionen/Stakob/Stellungnahmen/Stellungnahme-Covid-19).

Data on lactate dehydrogenase (LDH) levels, serum creatinine levels and thrombocyte counts were raised in certified routine laboratories. For calculation of EASIX, parameters obtained at the time of hospital admission were considered.

### Diagnosis, Supportive Care and Treatment

In Heidelberg, RNA was isolated from nasopharyngeal and oropharyngeal swab specimens using QIAGEN Kits (QIAGEN, Hilden, Germany) automated on the QIASymphony (DSP Virus/Pathogen mini Kits) or QIAcube (QIAamp Viral RNA mini Kits) devices and eluted in 115 μl elution buffer. RT-PCR was carried out using various reagent mixes – LightMix Modular SARS and Wuhan CoV E-gene, LightMix Modular SARS and Wuhan CoV N-gene, LightMix Modular Wuhan CoV RdRP-gene and LightMix Modular EAV RNA Extraction Control (as internal Control) from TIB MOLBIOL Syntheselabor GmbH (Berlin, Germany) and LightCycler Multiplex RNA Virus Master (Roche, Germany) – according to manufacturer’s instructions. RT-PCR was performed on LightCycler 480 oder 480 II (Roche, Germany).

In Ludwigshafen, the same protocol was used except that extraction was carried out using magnetic Nuclesens easyMag^®^ silica beads of Biomerieux followed by one-step qRT-PCR (SuperScript III Plantinum qRT-PCR Kit of ThermoFisher Scientific for qualitative and quantitative real-time PCR on Roche 480 II instruments. The analytical sensitivity was 100 copies/ml in both procedures. In Munich, laboratory confirmation of SARS-CoV-2 infection was done using the Abbott RealTime SARS-CoV-2 test kit (dual target Assay to detect the RdRp- and N-Gene), Abbott m2000sp extracting the probes and Abbott m2000rt for the RT-PCR. The analytical sensitivity was 100 copies/ml.

Criteria for initiation of mechanical ventilation were failure to maintain adequate ventilation or oxygenation in spite of high FiO2 delivery. Patients were treated with standard supportive care including antibiotic and antifungal therapy, whereas additional immunomodulatory therapy was inconsistently applied (azithromycin, hydroxychloroquine, tocilicumab, anakinra, prednisolone, maraviroc, remdesivir, Cytosorb™, plasmapheresis). Strategies of extracorporeal life support (extracorporeal CO2 elimination, veno-veno ECMO, veno-arterial ECMO) followed institutional policies. Routine CT scans of all patients were performed at hospital admission in all centers.

### Assessment of Cytokine Serum Levels

Serum samples were collected in gel tubes (S-Monovette^®^ Z-Gel, SARSTEDT AG & Co. KG, Nuembrecht, Germany) at the time SARS-CoV-2 testing and cryopreserved at −80°C. Serum levels of soluble thrombomodulin (sTM, sCD141), suppressor of tumorigenicity 2 (ST2), Angiopoietin-2 (Ang2), chemokine-X-C-ligand 8 (CXCL8, interleukin 8), CXCL9 (monokine induced by gamma interferon, MIG), interleukin-18 (IL18), interleukin-18 binding protein A (IL18BPa), and interferon-alpha (IFNα) were assessed by ELISA using commercial kits (DuoSet, R&D Systems, Wiesbaden, Germany) according to the manufacturer’s instructions as reported previously (19, 20).

### Statistics

Categorical data of patient characteristics were compared using the Fisher exact test. Continuous variables were compared applying the Kruskal-Wallis test. Endothelial Activation and Stress Index (EASIX) was calculated according to the formula: LDH [U/L] x creatinine [mg/dL]/thrombocytes [10^9 cells per L] (23, 24, 27).

Survival was calculated from the date of admission to last follow up or death of any cause. Patients alive were censored at the date of last contact. Patients who were alive without necessary ventilation were censored at the time of the last contact. In addition, time to severe course of disease was analyzed, defined as time without mechanical ventilation and/or death (V/D) until reference day +28. Survival curves were calculated using Kaplan-Meier estimates, the follow-up distribution was estimated using the reverse Kaplan-Meier method.

The primary endpoint, severe course of the disease defined as mechanical ventilation and/or death of any cause (V/D) until reference day +28 was analyzed using uni- and multivariable logistic regression models. For uni- and multivariable analysis of time to V/D and survival, Cox regression models were used. Confounders known to be associated with COVID-19 mortality (5, 6, 35) (age, gender, comorbidity) were used as covariates in the multivariable models. Predictive accuracy of EASIX was evaluated by the Brier score and the AUC, the area under the receiver operating characteristic (ROC) curve for severity of disease (36). For time-to-event analysis time-dependent versions of the Brier score and the AUC were used to measure the predictive performance of EASIX (37, 38). For illustration purposes, an optimal EASIX cut point with respect to the different endpoints was determined by generalized maximally selected statistics using Monte Carlo resampling (39). The calculated cut point >2 (2.03) could be used to define high-risk groups.

Calculations were done using IBM^®^ SPSS^®^ Statistics, Version 24.0.0 and R, version 3.6.3 together with R packages coin, version 1.3-1, ModelGood, version 1.0.9, pec, version 2019.11.03, and riskRegression, version 2020.02.05. All statistical tests were two-sided at a significance level of 5%. Odds ratios (OR) and hazard ratios (HR) were estimated with 95% confidence interval (95% CI).

## Results

### Patients

Between February 2020 and September 2020, 100 consecutive patients were enrolled onto the prospective registry study at the University of Heidelberg, whereas the validation cohort comprised 126 patients (Munich, n=88; Ludwigshafen, n=38). Patient characteristics are summarized in **Table 1**. The two cohorts were comparable in terms of gender and comorbidities, but patients from the prospective cohort were significantly older. Moreover, there were significant differences for the single EASIX parameters (higher LDH and platelets in the training cohort, higher creatinine in the validation cohort, resulting in a balanced EASIX ratio). Age positively correlated with both EASIX and LDH (Spearman-rho 0.316, p<0.001 for EASIX and 0.352, p<0.001 for LDH), whereas no correlation with age was found for creatinine and platelets. This association with age was similar in male and female patients.

**Table 1:** Patient characteristics.

	Training cohort n=100	Validation cohort n=126	*P*
**Median age (range, years)**	64 (23-91)	55 (16-87)	0.005
**Age**			0.002
<60 years	39 (39)	74 (59)	
≥60 years	61 (61)	52 (41)	
**Gender, n (%)**			0.999
Male	63 (63)	76 (60)	
Female	37 (37)	50 (40)	
**Comorbidities, n (%)**			
CVD (including arterial hypertension)	40 (40)	49 (39)	
Diabetes	10 (10)	15 (12)	
Chronic kidney disease	11 (11)	11 (9)	
Chronic lung disease	11 (11)	17 (13)	
Malignancy	10 (10)	9 (7)	
None	45 (45)	67 (53)	
**Comorbidities, n (%)**			0.503
Any	55 (55)	59 (47)	
None	45 (45)	67 (53)	
**Median LDH [U/L] (range)**	380 (123-1843)	280 (109-1112)	<0.001
**Median creatinine [mg/dL] (range)**	0.88 (0.46-6.20)	0.90 (0.58-8.80)	0.047
**Median thrombocytes [10^9 cells per L] (range)**	222 (96-691)	200 (28-873)	0.041
**Median EASIX (range)**	1.67 (0.32-19.09)	1.45 (0.33-151.6)	0.473

CVD, cardiovascular disease; LDH, lactate dehydrogenase; EASIX, endothelial activation and stress index.

### EASIX and Outcome of COVID-19 Patients in the Prospective Cohort

Within a median observation period of 61 days (95%CI 59-64 days), a total of 23 patients had V/D, including 13 deaths. EASIX showed a significant effect on V/D events within the observation period of 28 days in univariable (OR 3.4, 95%CI 1.8-6.3, p<0.001) and multivariable (OR 3.4, 95%CI 1.8-6.7, p<0.001) logistic regression analysis (**Table 2**).

**Table 2:** Uni- and multivariable analyses for endpoint severe course of the disease and survival in the training and validation cohort.

Training cohort, n=100	Severe course (V/D) until d+28*			Time to severe course (V/D) n=21 events**			Death n=13 events**			
**a) univariable**	OR (95% CI)	p		HR (95% CI)	p		HR (95% CI)	p		
EASIX (per 2 fold increase)	4.25 (1.93-9.32)	<0.001		2.2 (1.6-3.0)	<0.001		2.1 (1.4-3.1)	<0.001		
**b) multivariable**	OR (95% CI)	p		HR (95% CI)	p		HR (95% CI)	p		
EASIX (per 2 fold increase)	3.4 (1.8-6.7)	<0.001		2.0 (1.4-2.8)	<0.001		1.7 (1.0-2.9)	0.038		
age (per year)	1.06 (1.0-1.1)	0.013		1.1 (1.0-1.1)	0.003		1.1 (1.0-1.2)	0.001		
gender (male *vs*. female)	1.3 (0.3-5.1)	0.690		1.8 (0.7-5.0)	0.251		2.8 (0.6-12.0)	0.170		
any comorbidity (no *vs*. yes)	3.0 (0.8-11.1)	0.111		2.3 (0.8-6.7)	0.111		4.0 (1.0-16.7)	0.048		
**Validation cohort, n=126**	Severe course (V/D) until d+28*			Time to severe course (V/D) n=33 events**			Death n=12 events**		
**a) univariable**	OR (95% CI)		p	HR (95% CI)		p	HR (95% CI)		p
EASIX (per 2 fold increase)	6.18 (2.99-12.79)		<0.001	2.5 (1.9-3.4)		<0.001	2.2 (1.4-3.4)		0.001
**b) multivariable**	OR (95% CI)		p	HR (95% CI)		p	HR (95% CI)		p
EASIX (per 2 fold increase)	4.84 (2.11-11.07)		<0.001	2.3 (1.7-3.3)		<0.001	2.0 (1.1-3.6)		0.02
age (per year)	1.03 (0.98-1.07)		0.218	1.0 (1.0-1.1)		0.02	1.1 (1.0-1.1)		0.09
gender (male *vs*. female)	0.82 (0.23-2.94)		0.755	1.0 (0.5-2.4)		0.92	0.5 (0.1-1.9)		0.31
any comorbidity (no *vs*. yes)	1.34 (0.39-4.61)		0.640	1.2 (0.5-2.9)		0.64	0.5 (0.1-2.8)		0.43

*logistic regression analysis; **Cox regression analysis.

V/D, ventilation and/or death; EASIX, endothelial activation and stress index; HR, hazard ratio; CI, confidentiality interval.

**Figure 1:**
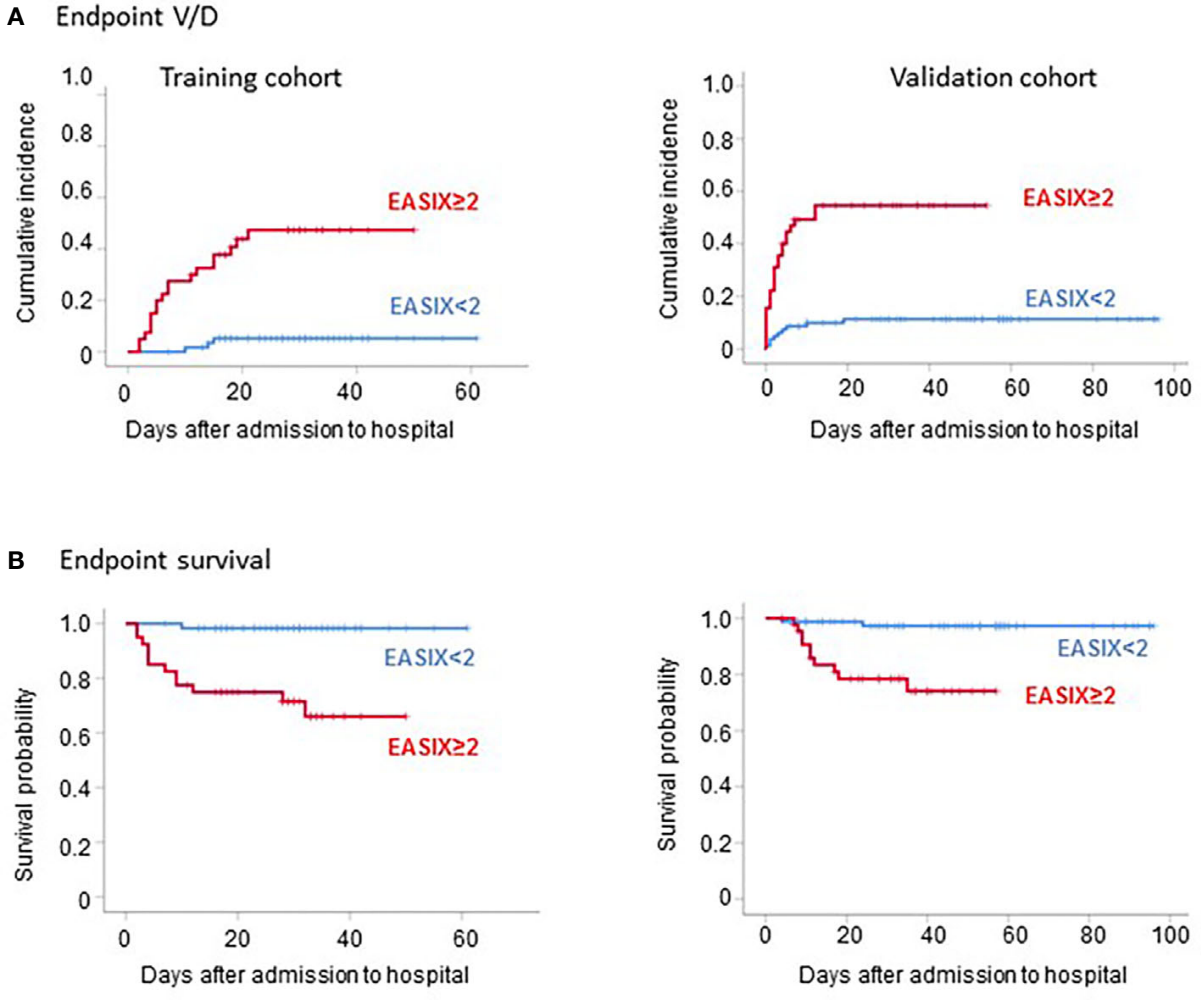
Outcome of COVID-19 patients according to EASIX. Outcome of COVID-19 patients according to EASIX (cut-off 2) in the training cohort (left panels) and the validation cohort (right panels). **(A)** Cumulative incidence of severe courses of disease (mechanical ventilation and/or death, V/D). **(B)** Kaplan-Meier plots of overall survival.

EASIX at admission was also a strong predictor of time to V/D (HR for a two-fold change 2.0, 95%CI 1.5-2.6, p<0.001) as well as survival (HR for a two-fold change 1.7, 95%CI 1.2-2.5, p=0.006). This strong effect was retained in multivariable analysis adjusting for age, gender, and comorbidities, although the reliability of this analysis is limited due to the small numbers of events (**Table 2**). An EASIX cut-off optimized by maximal selected log rank statistics was identified for both endpoints (V/D and death) to be at >2.0 (**Supplementary Figure 1**). Of note, only 3 of 21 V/D events and 1 of 13 deaths occurred among the patients who had an EASIX ≦2 (**Figure 1**). Patient characteristics according to EASIX are shown in **Supplementary Table 1**. Patients with EASIX>2 were older, predominantly male and more often had comorbidities. The expected differences (high LDH, high creatinine, low platelets for EASIX>2) were significant in all three single EASIX parameters (**Supplementary Table 1**).

We observed significantly higher EASIX values ad admission to hospital in male as compared to female patients. Nevertheless, patients with V/D events had significantly higher EASIX values in both gender subgroups (**Supplementary Figure 2A**). EASIX-log2 and EASIX>2 significantly predicted V/D events in both, male and female patients.

### EASIX and Outcome of COVID-19 Patients in the Validation Cohort

Within a median observation period of 41 days (IQR 24-56 days), a total of 33 patients had V/D in the validation cohort, including 12 deaths. Similar to the training cohort, EASIX was also significantly associated with V/D in the univariable logistic regression model (OR 6.2 (95%CI 3.0-12.8, p<0.001). Validation of the predictive impact of EASIX on V/D events was achieved by calculating the area under the ROC curve in the validation set with the model of the training cohort. We observed an AUC of 88.8% for the univariable and 87.9% for the multivariable model (**Supplementary Figure 3**).

Uni- and multivariable models confirmed the significant impact of EASIX on time to V/D and survival (**Table 2**). Lower prediction errors confirmed the predictive effect of EASIX: Integrated Brier score (IBS) (time day+28) for time to V/D: reference 0.175, validation cohort based on the model developed for the training cohort: 0.126.

Validation of the uni- and multivariable time-dependent models with the offset of the prospective cohort was performed using again the time-dependent Brier score. Lower prediction errors for prediction of V/D or survival were found both for uni- and multivariable models including EASIX (continuous variable) (**Supplementary Figure 4**).

Accordingly using the same EASIX cut-off as defined in the prospective cohort (≤2), the low-risk group in the validation cohort had a strongly reduced risk of V/D and death (9 of 33 V/D events and 2 of 12 deaths occurred among the patients who had an EASIX ≦2) (**Figure 1**). Similar to the prospective cohort, the high-risk EASIX group (>2) of the validation set was enriched for elderly male patients and those with comorbidities (**Supplementary Table 1**). Again, all three single EASIX parameters were significantly involved (**Supplementary Table 1**).

### EASIX and Endothelial and Inflammatory Biomarkers

**Figure 2:**
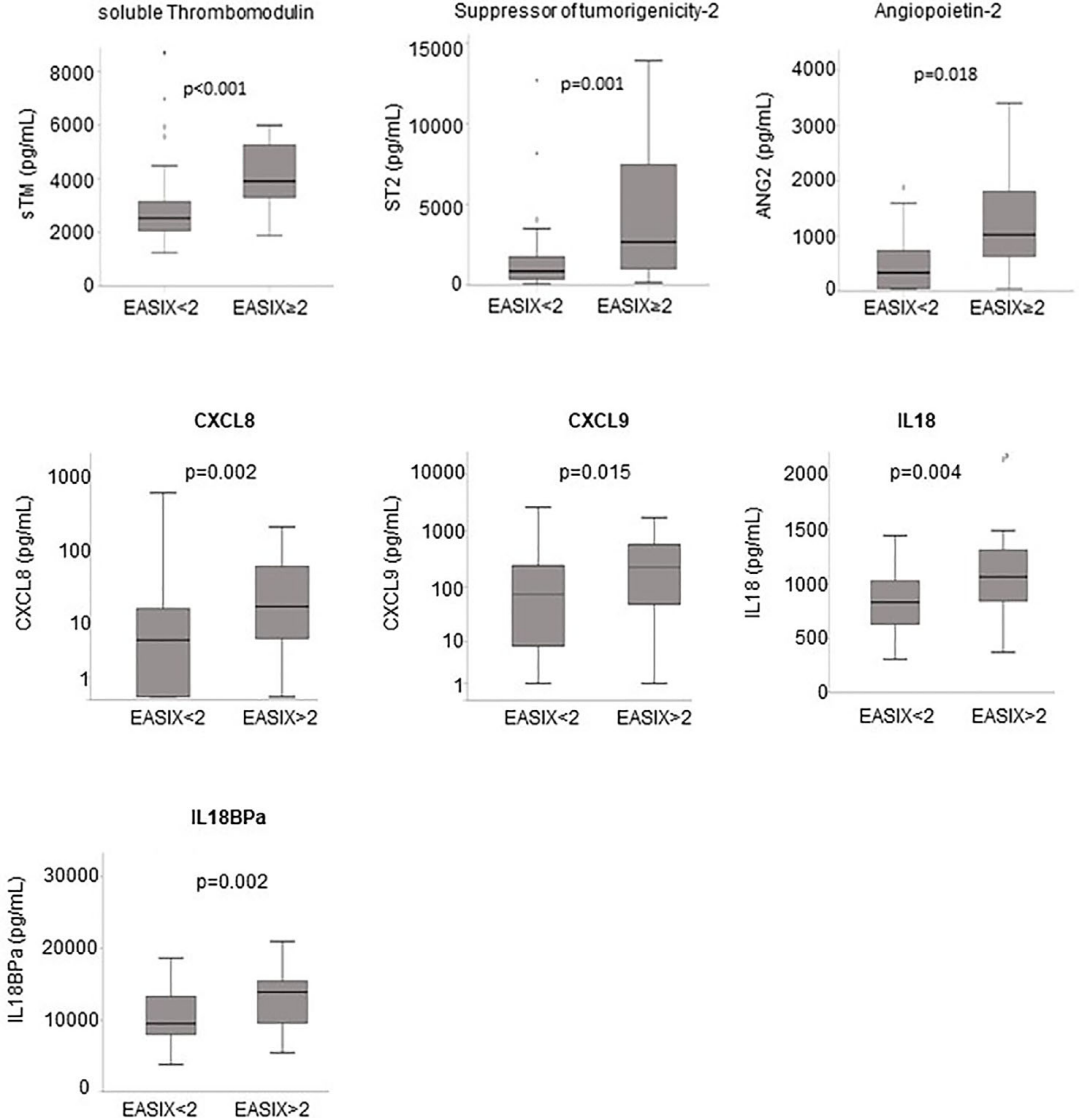
Endothelial markers and EASIX. Boxplots of serum levels of endothelial markers according to the EASIX cut-off: angiopoietin-2 (ANG2), suppressor of tumorigenicity-2 (ST2), soluble thrombomodulin (sTM), CXCL8 (interleukin-8), CXCL9 (monokine induced by gamma interferon, MIG), interleukin-18 (IL18) and IL18 binding protein A (IL18BPa). P-values for Kruskal-Wallis tests, n=87. Spearman-rho correlation coefficients with EASIX as continuous variable (n=87): ANG2 0.355, p < 0.001; sCD141 0.397, p < 0.001; ST2 0.397, p < 0.001; CXCL8 0.347, p < 0.001; IL-18 0.318, p=0.003; IL18BPa 0.260, p=0.015, CXCL9 0.453, p < 0.001.

Serum was only available for patients of the prospective cohort. Patients with V/D showed significantly higher serum levels of the endothelial markers ANG2, sCD141, ST2, and CXCL8 at admission (3.1-, 1.5-, 4.0- and 4.0-fold, respectively, **Table 3**). Similarly, the inflammatory markers CXCL9, IL18 and IL18BPa were also increased in patients with later severe disease courses (5.3-, 1.2- and 1.5-fold, respectively **Table 3**). EASIX>2 correlated significantly with increased serum levels at admission of both, endothelial and inflammatory markers (**Figure 2**). Interferon-alpha (IFNα) serum levels above the lower detection threshold (1 pg/ml) were found in only 16 of 86 patients, and no association was observed with EASIX or severe courses of the disease. Similar to EASIX, we found that age correlated with all serum markers except IFNα (n=87, Spearman-rho, p): ANG2 0.355, <0.001; sCD141 0.397, <0.001; ST2 0.397, <0.001; CXCL8 0.347, <0.001; IL-18 0.318, 0.003; IL18BPa 0.260, 0.015, CXCL9 0.453, <0.001; IFNα -0.009, 0.932. In addition, the higher level of endothelial distress in male patients shown by EASIX was mirrored by ANG2, sCD141 and ST2 (**Supplementary Figures 2B–D**).

**Table 3:** Endothelial and immune markers at hospital admission in the training cohort (total, n=83; no V/D, n=62; V/D, n=21).

pg/mL (IQR)	ANG2	sTM	ST2	CXCL8	CXCL9	IL18	IL18BPa	IFNα
no V/D	334 (700)	2529 (1098)	858 (1290)	6 (25)	61 (262)	867 (416)	9569 (5620)	0 (0)
V/D	1024 (1894)	3907 (2272)	3410 (7275)	24 (63)	323 (438)	1079 (512)	14495 (4663)	0 (2.2)
Fold increase	3.1	1.5	4.0	4.0	5.3	1.2	1.5	1
p	0.001	<0.001	<0.001	0.001	0.001	0.014	0.001	0.437

ANG2, angiopoietin-2; sTM, soluble thrombomodulin; ST2, suppressor of tumorigenicity-2, CXCL8, chemokine-X-C-ligand 8, (interleukin 8); CXCL9, chemokine-X-C-ligand 9, (monokine induced by gamma interferon, MIG); IL18, interleukin 18; IL18BPa, interleukin 18 binding protein A; IFNα, interferon-alpha; IQR, interquartile range (IQR=Q3-Q1); V/D, ventilation and/or death (until day+28 after admission).

## Discussion

This study reports EASIX as a validated predictor of mechanical ventilation and/or death of hospitalized COVID-19 patients.

Given the urgent clinical need of early distinction of unspectacular from severe COVID-19 courses, a large variety of prognostic markers have been proposed since the pandemic began in early 2020. These include, amongst others, high creatinine, high LDH, and low platelets (40–44). EASIX, however, amalgamates these markers into a score that distinguishes high- and low-risk patients with high accuracy: Whereas about half of those patients who present with EASIX ≥2 at admission will face a severe course and a mortality risk of 40%-60%, patients with admission EASIX <2 have a likelihood of severe complications of less than 15%, and less than 5% will die from the disease. This excellent selectivity of EASIX in COVID-19 might rely on the fact that it integrates biomarkers reflecting different mechanisms of endothelial dysfunction, thereby highlighting the endothelium as critical driver of COVID-19 pathogenesis (10–13).

To further elucidate the involvement of the endothelium in COVID-19 and its relation to EASIX, we have applied an endothelial biomarker panel to COVID-19, including angiopoietin-2 (ANG2), soluble thrombomodulin (sTM), suppressor of tumorigenicity-2 protein (ST2), and correlated them with EASIX. ANG2 antagonizes ANG1 at the Tie2 receptor and enhances vascular permeability. This cytokine was already shown to associate with severe COVID-19 courses (16). sTM is lost from surfaces of distressed endothelial cells where it usually mediates endothelial protective effects (45). ST2 is also produced by distressed endothelial cells and is a predictor of cardiovascular death (46, 47). All three serum markers were shown to associate with endothelial complications and outcome after allogeneic stem cell transplantation (alloSCT) (19, 48, 49). Here we demonstrate that these three endothelial markers are strongly increased in patients with severe disease courses. Similarly, high EASIX ratios (≥2) strongly correlated with high serum levels of endothelial markers. In this line of evidence, circulating endothelial cells (CECs) and high Angiopoietin-2 were found to associate with severity of COVID-19 (16, 50, 51). Accordingly, the ESC Working Group for Atherosclerosis and Vascular Biology, and the ESC Council of Basic Cardiovascular Science proposed that endothelial biomarkers and tests of function should be evaluated for their usefulness in the risk stratification of COVID-19 patients (52).

Due to the heterogeneity of endothelial response patterns it is difficult to define global or tissue specific endothelial distress markers that predict in all clinical settings. EASIX was developed to address this problem in allogeneic stem cell transplantation, where a variety of complications associating with non-relapse mortality represent endothelial complications. EASIX derives from diagnostic parameters associating with transplant-associated thrombotic microangiopathy (TAM) (21). High LDH, high creatinine and low platelets are the typical lab marker constellation in this microangiopathy, therefore we assessed if the ratio LDH*creatinine/platelets contains information on the endothelial system. Indeed, EASIX predicted TAM already prior to conditioning therapy, but it also predicted sinusoidal obstruction syndrome/veno-occlusive disease (SOS/VOD) (22), early fluid retention (26), and death after acute graft-versus-host disease (GVHD) (23). Recently, EASIX was also shown to predict survival of lower risk myelodysplastic syndromes (MDS) (27), which is a condition with a high risk of death from cardiovascular complications.

There is now good evidence that EASIX indeed represents systemic endothelial dysfunction. This is further underlined by the correlation of EASIX with other endothelial stress markers measured at the beginning of hospitalization, such as ANG2, ST2 und soluble thrombomodulin. Endotheliitis and cardiovascular vulnerability of COVID-19 patients led us to test if EASIX might help to prognosticate this disease as well.

We observed a gender-indifferent association of EASIX and LDH with age. Similarly, higher age strongly correlated with higher ANG2, ST2, and sCD141. In addition, male COVID-19 patients had significantly higher EASIX values, but also higher serum levels of ANG2, sCD141 and ST2. As neither age nor gender were associated with EASIX in other contexts (23, 24) we think that this reflects the higher vulnerability of elderly male endothelial cells towards viral challenge.

In summary, EASIX is a reliable and validated early predictor of COVID19 outcome. Specifically, EASIX≥2 appears to be a valuable and easy-to-access tool to segregate patients in need for intensive surveillance because of high risk of severe complications and mortality from those who have a very low risk of a fatal outcome. This is of tremendous clinical importance since in the absence of an effective causal treatment for COVID-19 and with limited intensive care capacities, identification of markers reliably predicting the course of infection may help in efficient resource allocation during the pandemic.

Moreover, the results of our study emphasize the importance of endothelial damage as a key factor in COVID-19 pathogenesis and may help deciphering disease biology. Further understanding of endothelial involvement may provide a rationale for interventions supporting endothelial cell integrity as part of clinical management of SARS-CoV-2 infected patients.

## Data Availability Statement

The raw data supporting the conclusions of this article will be made available by the authors, without undue reservation.

## Ethics Statement

The studies involving human participants were reviewed and approved by University Hospital Heidelberg Ethics Committee. The patients/participants provided their written informed consent to participate in this study.

## Author Contributions

All authors listed have made a substantial, direct, and intellectual contribution to the work and approved it for publication.

## Conflict of Interest

The authors declare that the research was conducted in the absence of any commercial or financial relationships that could be construed as a potential conflict of interest.

## References

[1] Lauer SA Grantz KH Bi Q Jones FK Zheng Q Meredith HR. The Incubation Period of Coronavirus Disease 2019 (Covid-19) From Publicly Reported Confirmed Cases: Estimation and Application. Ann Intern Med (2020) 172(9):577–82. 10.7326/M20-0504 PMC7081172 32150748

[2] Wang D Hu B Hu C Zhu F Liu X Zhang J. Clinical Characteristics of 138 Hospitalized Patients With 2019 Novel Coronavirus-Infected Pneumonia in Wuhan, China. JAMA (2020) 323(11):1061–9. 10.1001/jama.2020.1585 PMC7042881 32031570

[3] Guzik TJ Mohiddin SA Dimarco A Patel V Savvatis K Marelli–Berg FM. Covid-19 and the Cardiovascular System: Implications for Risk Assessment, Diagnosis, and Treatment Options. Cardiovasc Res (2020) 116(10):1666–87. 10.1093/cvr/cvaa106 PMC7197627 32352535

[4] Shi S Qin M Shen B Cai Y Liu T Yang F. Association of Cardiac Injury With Mortality in Hospitalized Patients With Covid-19 in Wuhan, China. JAMA Cardiol (2020) 5(7):802–10. 10.1001/jamacardio.2020.0950 PMC7097841 32211816

[5] Yang J Zheng Y Gou X Pu K Chen Z Guo Q. Prevalence of Comorbidities and Its Effects in Coronavirus Disease 2019 Patients: A Systematic Review and Meta-Analysis. Int J Infect Dis (2020) 94:91–5. 10.1016/j.ijid.2020.03.017 PMC7194638 32173574

[6] Zhou F Yu T Du R Fan G Liu Y Liu Z. Clinical Course and Risk Factors for Mortality of Adult Inpatients With COVID-19 in Wuhan, China: A Retrospective Cohort Study. Lancet (2020) 395(10229):1054–62. 10.1016/S0140-6736(20)30566-3 PMC7270627 32171076

[7] Verdoni L Mazza A Gervasoni A Martelli L Ruggeri M Ciuffreda M. An Outbreak of Severe Kawasaki-Like Disease at the Italian Epicentre of the SARS-CoV-2 Epidemic: An Observational Cohort Study. Lancet (2020) 395(10239):1771–8. 10.1016/S0140-6736(20)31103-X PMC7220177 32410760

[8] Viner RM Whittaker E. Kawasaki-Like Disease: Emerging Complication During the COVID-19 Pandemic. Lancet (2020) 395(10239):1741–3. 10.1016/S0140-6736(20)31129-6 PMC7220168 32410759

[9] Bikdeli B Madhavan MV Jimenez D Chuich T Dreyfus I Driggin E. Covid-19 and Thrombotic or Thromboembolic Disease: Implications for Prevention, Antithrombotic Therapy, and Follow-Up: JACC State-of-the-Art Review. J Am Coll Cardiol (2020) 75(23):2950–73. 10.1016/j.jacc.2020.04.031 PMC7164881 32311448

[10] Escher R Breakey N Lammle B. Severe COVID-19 Infection Associated With Endothelial Activation. Thromb Res (2020) 190:62. 10.1016/j.thromres.2020.04.014 32305740 PMC7156948

[11] Paniz-Mondolfi A Bryce C Grimes Z Gordon RE Reidy J Lednicky J. Central Nervous System Involvement by Severe Acute Respiratory Syndrome Coronavirus-2 (SARS-Cov-2). J Med Virol (2020) 92(7):699–702. 10.1002/jmv.25915 32314810 PMC7264598

[12] Teuwen LA Geldhof V Pasut A Carmeliet P. Covid-19: The Vasculature Unleashed. Nat Rev Immunol (2020) 20(7):389–91. 10.1038/s41577-020-0343-0 PMC7240244 32439870

[13] Varga Z Flammer AJ Steiger P Haberecker M Andermatt R Zinkernagel AS. Endothelial Cell Infection and Endotheliitis in COVID-19. Lancet (2020) 35(10234):1417–8. 10.1016/S0140-6736(20)30937-5 PMC7172722 32325026

[14] Hoffmann M Kleine-Weber H Schroeder S Kruger N Herrler T Erichsen S. SARS-Cov-2 Cell Entry Depends on ACE2 and TMPRSS2 and Is Blocked by a Clinically Proven Protease Inhibitor. Cell (2020) 181(2):271–80.e8. 10.1016/j.cell.2020.02.052 32142651 PMC7102627

[15] Ciceri F Beretta L Scandroglio AM Colombo S Landoni G Ruggeri A. Microvascular COVID-19 Lung Vessels Obstructive Thromboinflammatory Syndrome (MicroCLOTS): An Atypical Acute Respiratory Distress Syndrome Working Hypothesis. Crit Care Resusc (2020)22(3):284. 32294809 10.51893/2020.2.pov2 PMC10692450

[16] Smadja DM Guerin CL Chocron R Yatim N Boussier J Gendron N. Angiopoietin-2 as a Marker of Endothelial Activation Is a Good Predictor Factor for Intensive Care Unit Admission of COVID-19 Patients. Angiogenesis (2020) 23(4):611–20. 10.1007/s10456-020-09730-0 PMC7250589 32458111

[17] Campbell CM Kahwash R. Will Complement Inhibition be the New Target in Treating Covid-19 Related Systemic Thrombosis? Circulation (2020). 10.1161/CIRCULATIONAHA.120.047419 32271624

[18] Liu PP Blet A Smyth D Li H. The Science Underlying COVID-19: Implications for the Cardiovascular System. Circulation (2020) 142(1):68–78. 10.1161/CIRCULATIONAHA.120.047549 32293910

[19] Luft T Dietrich S Falk C Conzelmann M Hess M Benner A. Steroid-Refractory GVHD: T-Cell Attack Within a Vulnerable Endothelial System. Blood (2011) 118(6):1685–92. 10.1182/blood-2011-02-334821 21636856

[20] Zeisbrich M Becker N Benner A Radujkovic A Schmitt K Beimler J. Transplant-Associated Thrombotic Microangiopathy Is an Endothelial Complication Associated With Refractoriness of Acute GvHD. Bone Marrow Transplant (2017) 52(10):1399–405. 10.1038/bmt.2017.119 28650448

[21] Ruutu T Barosi G Benjamin RJ Clark RE George JN Gratwohl A. Diagnostic Criteria for Hematopoietic Stem Cell Transplant-Associated Microangiopathy: Results of a Consensus Process by an International Working Group. Haematologica (2007) 92(1):95–100. 10.3324/haematol.10699 17229640

[22] Jiang S Penack O Terzer T Schult D Majer–Lauterbach J Radujkovic A. Predicting Sinusoidal Obstruction Syndrome After Allogeneic Stem Cell Transplantation With the EASIX Biomarker Panel. Haematologica (2020) 92(1):95–100. 10.3324/haematol.2019.238790 PMC7849560 31974195

[23] Luft T Benner A Jodele S Dandoy CE Storb R Gooley T. EASIX in Patients With Acute Graft-Versus-Host Disease: A Retrospective Cohort Analysis. Lancet Haematol (2017) 4(9):e414–e23. 10.1016/S2352-3026(17)30108-4 28733186

[24] Luft T Benner A Terzer T Jodele S Dandoy CE Storb R. EASIX and Mortality After Allogeneic Stem Cell Transplantation. Bone Marrow Transplant (2020) 55(3):553–61. 10.1038/s41409-019-0703-1 PMC8082940 31558788

[25] Shouval R Fein JA Shouval A Danylesko I Shem–Tov N Zlotnik M. External Validation and Comparison of Multiple Prognostic Scores in Allogeneic Hematopoietic Stem Cell Transplantation. Blood Adv (2019) 3(12):1881–90. 10.1182/bloodadvances.2019032268 PMC6595255 31221661

[26] Varma A Rondon G Srour SA Chen J Ledesma C Champlin RE. Endothelial Activation and Stress Index (EASIX) at Admission Predicts Fluid Overload in Recipients of Allogeneic Stem Cell Transplantation. Biol Blood Marrow Transplant (2020) 3(12):1881–90. 10.1016/j.bbmt.2020.01.028 32045652

[27] Merz A Germing U Kobbe G Kaivers J Jauch A Radujkovic A. EASIX for Prediction of Survival in Lower-Risk Myelodysplastic Syndromes. Blood Cancer J (2019) 9(11):85. 10.1038/s41408-019-0247-z 31712595 PMC6848148

[28] Song GY Jung SH Kim K Kim SJ Yoon SE Lee HS. Endothelial Activation and Stress Index (EASIX) Is a Reliable Predictor for Overall Survival in Patients With Multiple Myeloma. BMC Cancer (2020) 20(1):803. 10.1186/s12885-020-07317-y 32831058 PMC7446202

[29] Dietrich S Falk CS Benner A Karamustafa S Hahn E Andrulis M. Endothelial Vulnerability and Endothelial Damage Are Associated With Risk of Graft-Versus-Host Disease and Response to Steroid Treatment. Biol Blood Marrow Transplant (2013) 19(1):22–7. 10.1016/j.bbmt.2012.09.018 23041600

[30] Dai H Rachakonda SP Penack O Blau IW Blau O Radujkovic A. Polymorphisms in CXCR3 Ligands Predict Early CXCL9 Recovery and Severe Chronic GVHD. Blood Cancer J (2021) 11(2):42. 10.1038/s41408-021-00434-2 33640906 PMC7914250

[31] Radujkovic A Kordelas L Bogdanov R Muller–Tidow C Beelen DW Dreger P. Interleukin-18 and Hematopoietic Recovery After Allogeneic Stem Cell Transplantation. Cancers (Basel) (2020) 12(10):2789. 10.3390/cancers12102789 PMC7601738 32998441

[32] Radujkovic A Kordelas L Dai H Schult D Majer-Lauterbach J Beelen D. Interleukin-18 and Outcome After Allogeneic Stem Cell Transplantation: A Retrospective Cohort Study. EBioMedicine (2019) 49:202–12. 10.1016/j.ebiom.2019.10.024 PMC6945194 31680001

[33] Contoli M Papi A Tomassetti L Rizzo P Vieceli Dalla Sega F Fortini F. Blood Interferon-Alpha Levels and Severity, Outcomes, and Inflammatory Profiles in Hospitalized Covid-19 Patients. Front Immunol (2021) 12:648004. 10.3389/fimmu.2021.648004 33767713 PMC7985458

[34] Pine AB Meizlish ML Goshua G Chang C-H Zhang H Bishai J. Circulating Markers of Angiogenesis and Endotheliopathy in COVID-19. Pulm Circ (2020) 10(4):2045894020966547. 10.1177/2045894020966547 33282193 PMC7691906

[35] Shi Y Yu X Zhao H Wang H Zhao R Sheng J. Host Susceptibility to Severe COVID-19 and Establishment of a Host Risk Score: Findings of 487 Cases Outside Wuhan. Crit Care (2020) 24(1):108. 10.1186/s13054-020-2833-7 32188484 PMC7081524

[36] Gerds TA Cai T Schumacher M. The Performance of Risk Prediction Models. Biom J (2008) 50(4):457–79. 10.1002/bimj.200810443 18663757

[37] Gerds TA Schumacher M. Consistent Estimation of the Expected Brier Score in General Survival Models With Right-Censored Event Times. Biom J (2006) 48(6):1029–40. 10.1002/bimj.200610301 17240660

[38] Heagerty PJ Lumley T Pepe MS. Time-Dependent ROC Curves for Censored Survival Data and a Diagnostic Marker. Biometrics (2000) 56(2):337–44. 10.1111/j.0006-341X.2000.00337.x 10877287

[39] Hothorn T Zeileis A. Generalized Maximally Selected Statistics. Biometrics (2008) 64(4):1263–9. 10.1111/j.1541-0420.2008.00995.x 18325074

[40] Ghahramani S Tabrizi R Lankarani KB Kashani SMA Rezaei S Zeidi N. Laboratory Features of Severe *vs*. Non-Severe COVID-19 Patients in Asian Populations: A Systematic Review and Meta-Analysis. Eur J Med Res (2020) 25(1):30. 10.1186/s40001-020-00432-3 32746929 PMC7396942

[41] Ou M Zhu J Ji P Li H Zhong Z Li B. Risk Factors of Severe Cases With COVID-19: A Meta-Analysis. Epidemiol Infect (2020) 148:e175. 10.1017/S095026882000179X 32782035 PMC7438625

[42] Panteghini M. Lactate Dehydrogenase: An Old Enzyme Reborn as a COVID-19 Marker (and Not Only). Clin Chem Lab Med (2020)58(12):1979–81. 10.1515/cclm-2020-1062 32829312

[43] Salamanna F Maglio M Landini MP Fini M. Platelet Functions and Activities as Potential Hematologic Parameters Related to Coronavirus Disease 2019 (Covid-19). Platelets (2020) 31(5):627–32. 10.1080/09537104.2020.1762852 32397915

[44] Wendel Garcia PD Fumeaux T Guerci P Heuberger DM Montomoli J Roche–Campo F. Prognostic Factors Associated With Mortality Risk and Disease Progression in 639 Critically Ill Patients With COVID-19 in Europe: Initial Report of the International RISC-19-ICU Prospective Observational Cohort. EClinicalMedicine (2020) 25:100449. 32838231 10.1016/j.eclinm.2020.100449 PMC7338015

[45] Nurnberger W Michelmann I Burdach S Gobel U. Endothelial Dysfunction After Bone Marrow Transplantation: Increase of Soluble Thrombomodulin and PAI-1 in Patients With Multiple Transplant-Related Complications. Ann Hematol (1998) 76(2):61–5. 10.1007/s002770050364 9540759

[46] Bartunek J Delrue L Van Durme F Muller O Casselman F De Wiest B. Nonmyocardial Production of ST2 Protein in Human Hypertrophy and Failure Is Related to Diastolic Load. J Am Coll Cardiol (2008) 52(25):2166–74. 10.1016/j.jacc.2008.09.027 PMC2637465 19095135

[47] Pascual-Figal DA Januzzi JL. The Biology of ST2: The International St2 Consensus Panel. Am J Cardiol (2015) 115(7 Suppl):3B–7B. 10.1016/j.amjcard.2015.01.034 25665766

[48] Andrulis M Dietrich S Longerich T Koschny R Burian M Schmitt-Gräf A. Loss of Endothelial Thrombomodulin Predicts Response to Steroid Therapy and Survival in Acute Intestinal Graft-Versus-Host Disease. Haematologica (2012) 97(11):1674–7. 10.3324/haematol.2011.061051 PMC3487439 22689672

[49] Vander Lugt MT Braun TM Hanash S Ritz J Ho VT Anthin JH. ST2 as a Marker for Risk of Therapy-Resistant Graft-Versus-Host Disease and Death. N Engl J Med (2013) 369(6):529–39. 10.1056/NEJMoa1213299 PMC3943357 23924003

[50] Guervilly C Burtey S Sabatier F Cauchois R Lano G Abdili E. Circulating Endothelial Cells as a Marker of Endothelial Injury in Severe Covid-19. J Infect Dis (2020) 222(11):1789–93. 10.1093/infdis/jiaa528 PMC7454721 32812049

[51] Mancuso P Gidaro A Gregato G Reveane A Cremonesi P Quarna J. Circulating Endothelial Progenitors are Increased in Covid-19 Patients and Correlate With SARS-CoV-2 RNA in Severe Cases. J Thromb Haemost (2020) 18(10):2744–50. 10.1101/2020.04.29.20085878 PMC7436444 32762140

[52] Evans PC Ed Rainger G Mason JC Guzik TJ Osto E Stamataki Z. Endothelial Dysfunction in COVID-19: A Position Paper of the ESC Working Group for Atherosclerosis and Vascular Biology, and the ESC Council of Basic Cardiovascular Science. Cardiovasc Res (2020) 116(14):2177–84. 10.1093/cvr/cvaa230 PMC7454368 32750108

