# Supplementary material for: EASIX for Prediction of Outcome in Hospitalized SARS-CoV-2 Infected Patients
Source: Front Immunol. 2021 Jun 23;12:634416. doi: 10.3389/fimmu.2021.634416 (PMC8261154; doi:10.3389/fimmu.2021.634416)

Table S1. Patient characteristics according to EASIX

	Training cohort n=100			Validation cohort n=126		
	**EASIX ≥2 n=40**	**EASIX <2 n=60**	***P***	**EASIX ≥2 n=45**	**EASIX <2 n=81**	***P***
**Median age (range, years)**	69 (31-85)	61 (23-90)	0.007	68 (20-85)	50 (16-87)	<0.001
**Age, n (%)**			0.036			<0.001
<60 years	10 (25)	28 (47)		12 (27)	62 (77)	
≥60 years	30 (75)	32 (53)		33 (73)	19 (23)	
**Gender, n (%)**			0.002			0.001
Male	32 (80)	29 (48)		37 (82)	42 (52)	
Female	8 (20)	31 (52)		8 (18)	39 (48)	
**Comorbidities, n (%)**						
CVD (including arterial hypertension)	21 (53)	19 (32)		29 (64)	20 (24)	
Diabetes	3 (8)	5 (8)		7 (16)	8 (10)	
Chronic kidney disease	3 (8)	5 (8)		8 (18)	3 (12)	
Chronic lung disease	6 (15)	5 (8)		7 (16)	10 (12)	
Malignancy	5 (13)	4 (7)		6 (13)	3 (4)	
None	15 (38)	30 (50)		12 (27)	55 (68)	
**Comorbidities, n (%)**			0.224			<0.001
Any	25 (63)	30 (50)		33 (73)	26 (32)	
None	15 (37)	30 (50)		12 (27)	55 (68)	
**Median LDH [U/L] (range)**	514 (213-1843)	333 (123-772)	<0.001	478 (165-1112)	237 (109-873)	<0.001
**Median creatinine [mg/dL] (range)**	1.03 (0.54-6.20)	0.78 (0.46-1.57)	<0.001	1.04 (0.58-8.80)	0.86 (0.60-1.90)	<0.001
**Median thrombocytes [10^9 cells per L] (range)**	196 (96-444)	242 (103-691)	0.002	154 (28-422)	216 (124-873)	<0.001
**Median EASIX (range)**	2.64 (2.04-19.09)	1.15 (0.32-1.97)	<0.001	3.26 (2.03-14.02)	0.94 (0.33-1.99)	<0.001

CVD, cardiovascular disease; LDH, lactate dehydrogenase; EASIX, endothelial activation and stress index.

Figure S1: Cut-point selection for EASIX in the training cohort, maximal selected log rank statistics.

Endpoint time to mechanical ventilation or death (V/D)

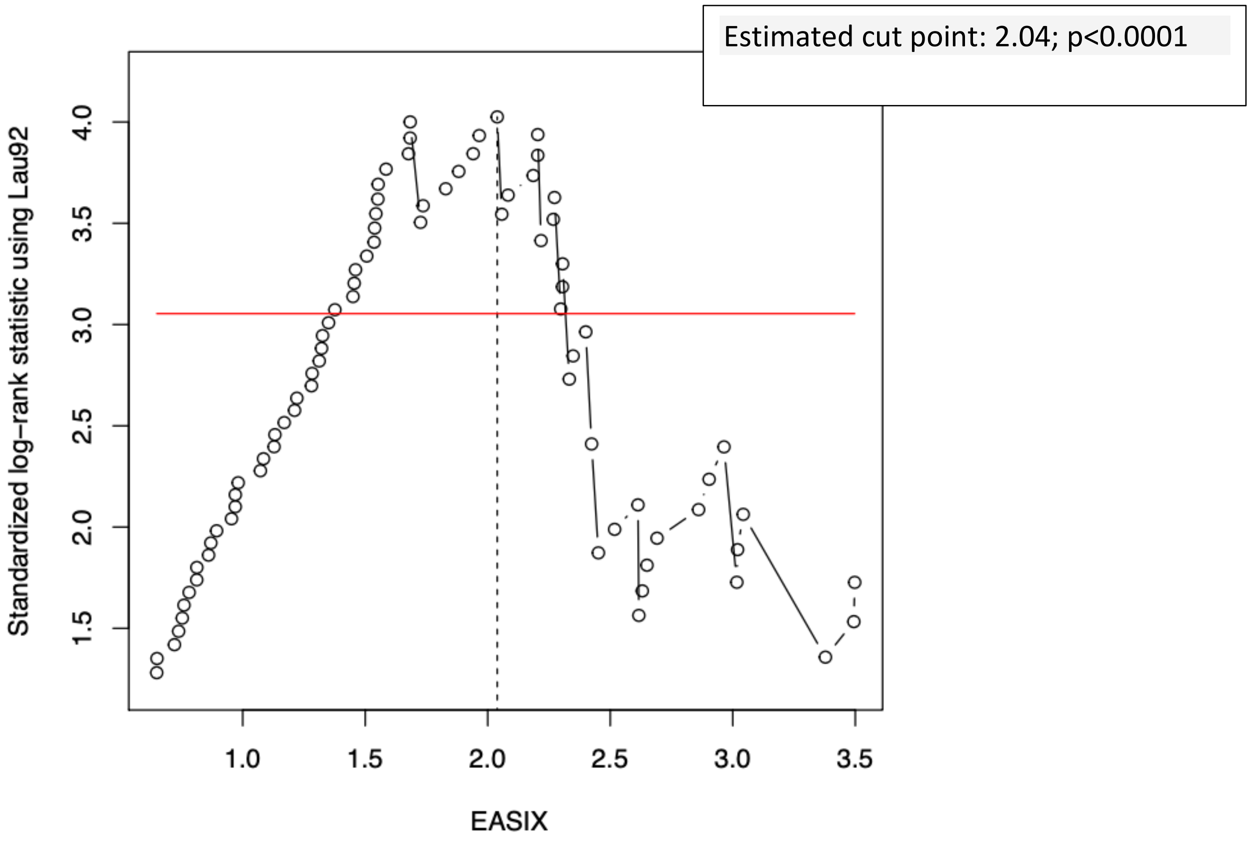

Figure S2: EASIX and endothelial markers in male and female patients

V/D, severe clinical course: ventilation and/or death.
p-values of Kruskal-Wallis tests.

A) EASIX

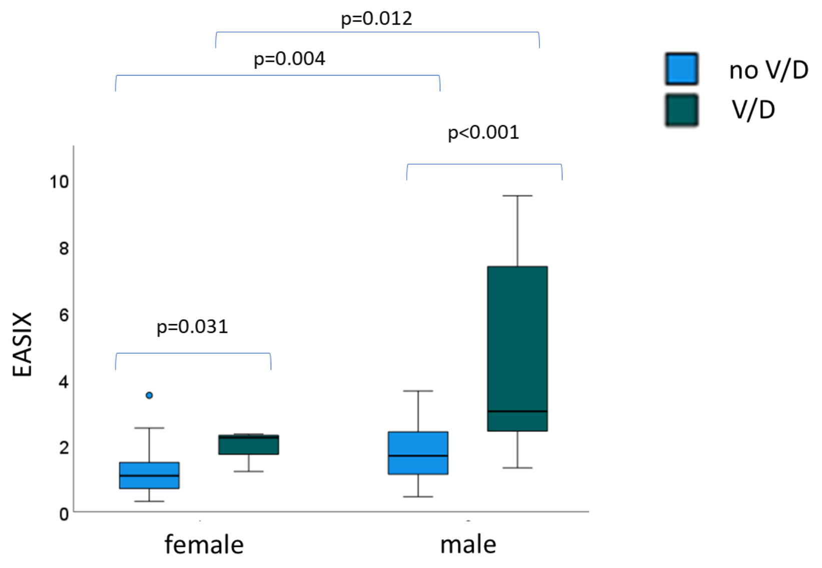

B) Angiopoietin-2 (ANG2)

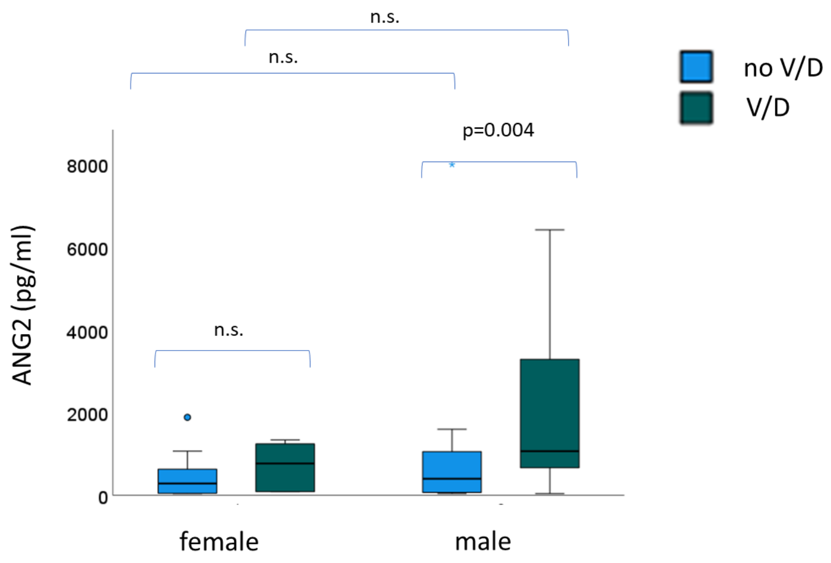

C) Soluble thrombomodulin (sCD141)

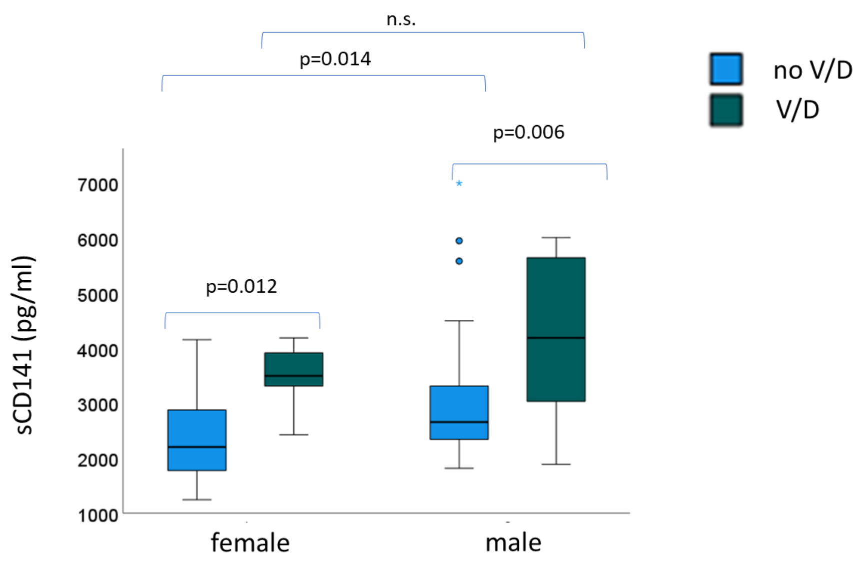

D) Suppressor of tumorigenicity (ST2)

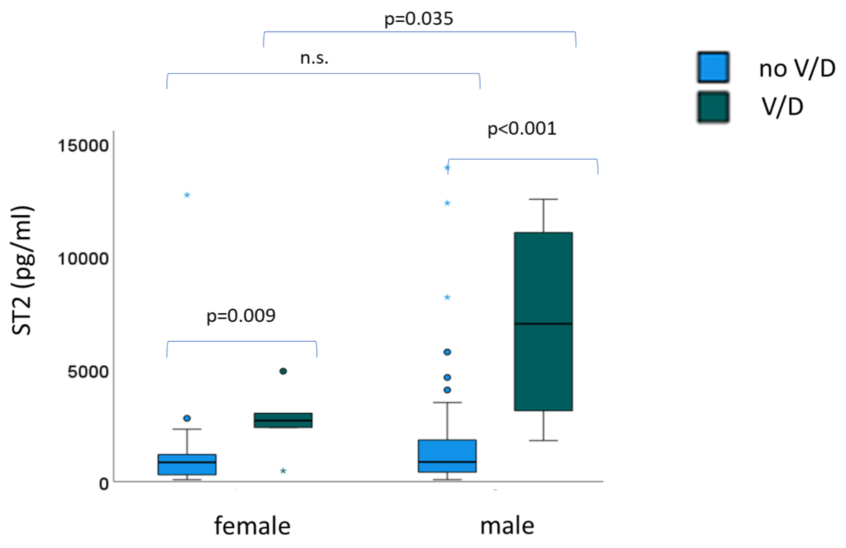

Figure S3: Validation of the uni- and multivariable logistic regression model of the training cohort using receiver operating characteristics (ROC) for endpoint mechanical ventilation and/or death (V/D) in the validation cohort.

Both models predict V/D events with high discrimination ability (area under the curve (AUC) above 85%.

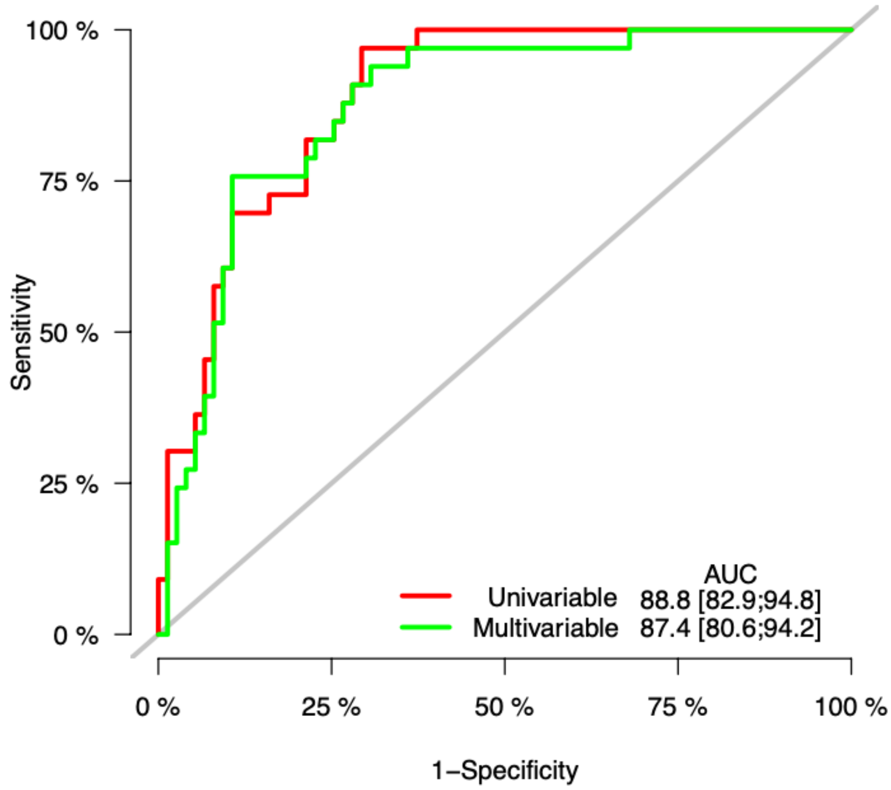

Figure S4: Validation of the predictive impact of EASIX on outcome of COVID-19

Prediction errors (time-dependent Brier scores (IBS) for the validation cohort with off-set training cohort) are shown for uni- and multivariable models for endpoints mechanical ventilation and/or death (V/D) and survival. Black lines: reference models (null model). Red lines (univariable) and green lines (multivariable) below the reference indicates model validation.

A) Endpoint V/D: IBS (time=day+28): reference 0.175, univariable 0.150, multivariable 0.151

B) Endpoint survival: IBS (time=day+92): reference 0.075, univariable 0.064, multivariable 0.070

A)

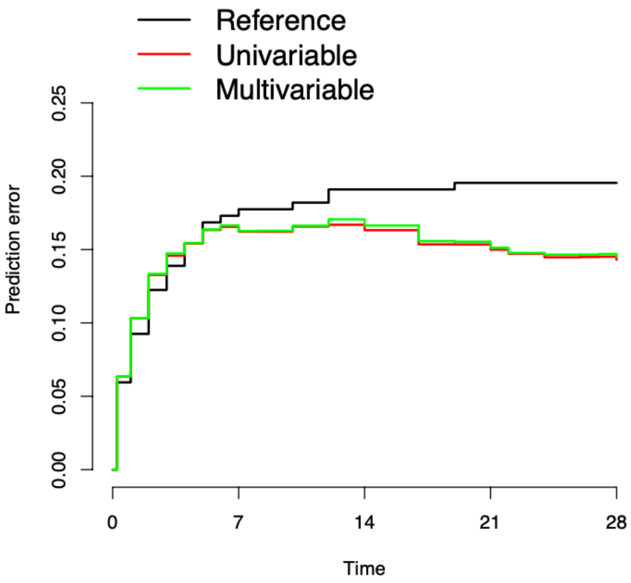

B)

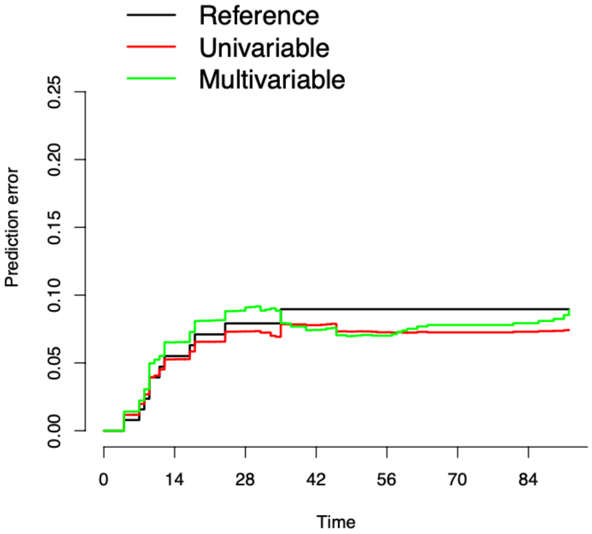

Supplement: Supplementary file 1 [file DataSheet_1.pdf]
